# Acute Achilles Tendon Ruptures Treated With Endoscopic Flexor Hallucis Longus Transfer

**DOI:** 10.1016/j.eats.2023.07.013

**Published:** 2023-10-11

**Authors:** Michail Kotsapas, Christos Koukos, Apostolos Polyzos, Symeon Naoum, Christos Koutserimpas, Alexandros Eleftheropoulos

**Affiliations:** a Department of Orthopaedics and Traumatology, Naoussa’s General Hospital, Naoussa, Greece; b Department of Special Sports Surgery, St Josef Krankenhaus, Wuppertal, Germany; c Department of Orthopaedics and Traumatology, 251 Hellenic Air Force General Hospital of Athens, Athens, Greece; d Department of Surgical Anatomy, Medical School of Athens, National and Kapodistrian University of Athens, Athens, Greece

## Abstract

The Achilles tendon represents the most commonly ruptured tendon of the human body. Numerous studies have evaluated, throughout the years, management options regarding acute Achilles tendon rupture. Minimally invasive techniques have recently gained more popularity. Endoscopic flexor hallucis longus tendon transfer has been mainly described as a treatment method for neglected Achilles tendon ruptures. However, it has recently been described as an applicable treatment option for acute Achilles tendon ruptures as well. Nevertheless, this procedure is technically quite demanding and should be performed by experienced surgeons. This technical note thoroughly describes the endoscopic flexor hallucis longus transfer technique for acute Achilles tendon ruptures, focusing on the most important technical aspects, thus attempting to simplify and render this procedure more widely accepted.

Acute Achilles tendon rupture (AATR) most commonly affects middle-aged men^1^ and sometimes may result in significant morbidity.^2^ AATR management remains controversial because there is no consensus regarding optimal treatment, even though numerous high-quality studies have been published. Both nonoperative management and operative management are acceptable; the main advantages of operative treatment are tendon length and strength restoration, as well as lower rerupture rates. The main drawbacks of surgical treatment consist of operation-related complications, including infection, scarring, and nerve injury.^3^

Endoscopic flexor hallucis longus (FHL) tendon transfer represents an operative technique that has mainly been used in low-demand patients with neglected Achilles tendon (AT) ruptures.^4^ Nevertheless, it was recently characterized as a safe and reliable treatment alternative in patients with AATR,^1^ even high-demand patients, such as professional athletes.^5^

In our institution, endoscopic FHL transfer is used as a treatment option for fit patients. A meticulous technique description is provided, as well as tips and tricks for performing this minimally invasive technique for the treatment of AATR (Video 1).

## Surgical Technique

**Fig 1:**
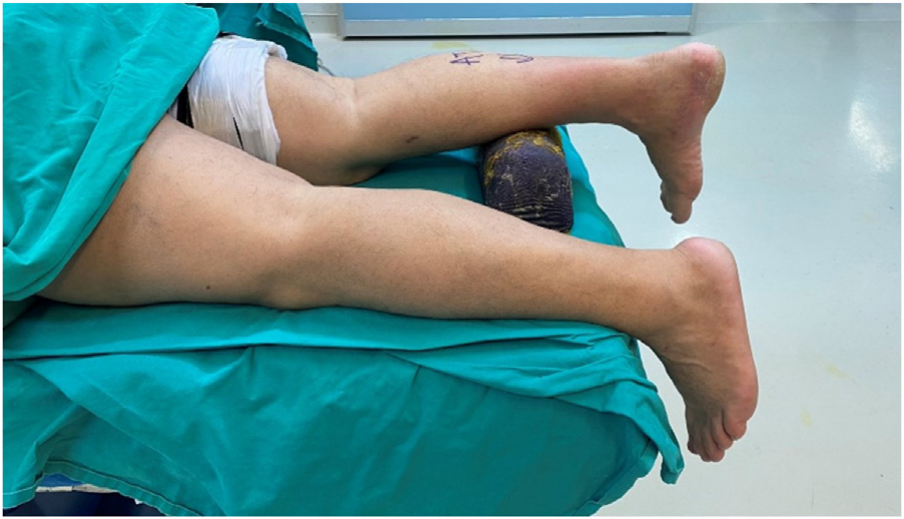
Patient positioning. Hindfoot endoscopy is performed with the patient placed in the prone position, with the foot hanging over the edge of the operating table; a soft pad is placed under the ipsilateral calf. A tourniquet (optional) is applied at thigh level and is inflated up to a maximum of 300 mm Hg.

The procedure may be performed with the patient under general or spinal anesthesia. The patient is placed in the prone position, with the foot hanging over the edge of the operating table; a soft pad is placed under the ipsilateral calf and another is placed under the contralateral pelvis to reduce external rotation of the ipsilateral limb. A tourniquet is applied at thigh level (Fig 1).

**Fig 2:**
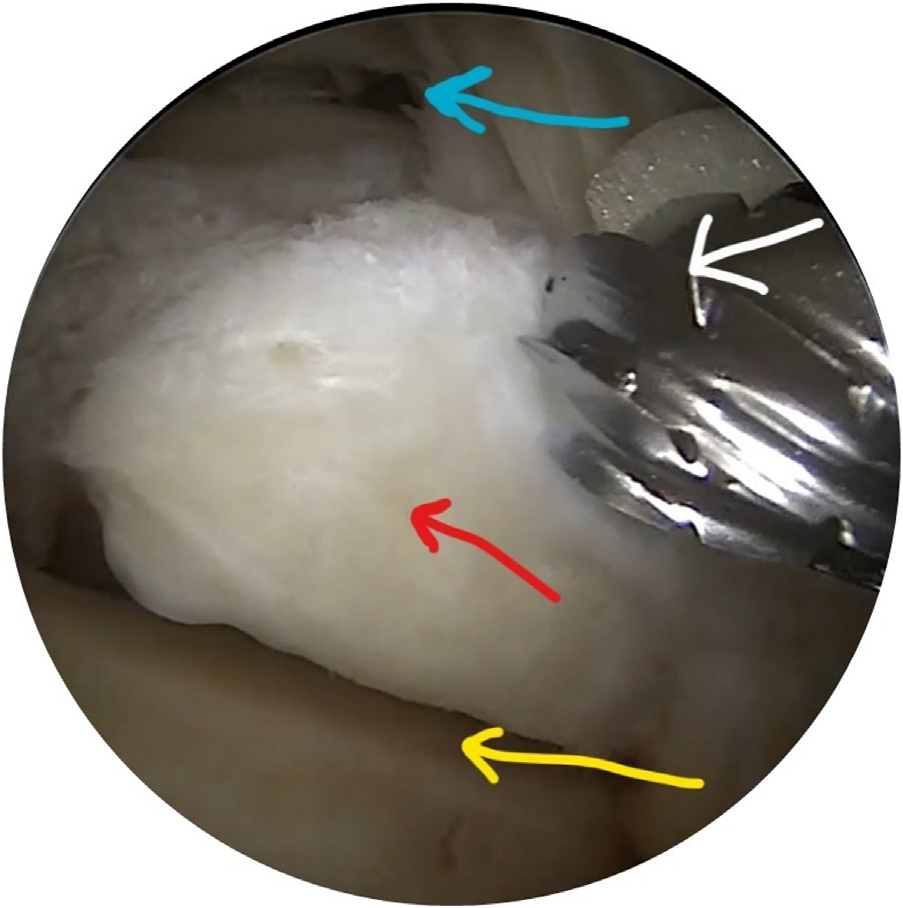
After debridement, some important anatomic landmarks may be identified. The scope is inserted through the posterolateral (viewing) portal, while an acromionizer is inserted through the posteromedial (working) portal. The blue arrow indicates the ankle joint; red arrow, os trigonum; and yellow arrow, subtalar joint. The white arrow indicates the acromionizer used to excise the os trigonum in this patient to facilitate access to the flexor hallucis longus tendon, which is located behind the acromionizer and the blue arrow.

Two portals are established at the level of the tip of the lateral malleolus: a posterolateral (viewing) portal and a posteromedial (working) portal.^6^ The posterolateral portal is made through a stab incision just lateral to the torn AT, and a 4.0-mm 30° arthroscope (Arthrex, Naples, FL) is inserted. Then, the posteromedial portal is established just medial to the torn AT. After cautious debridement with a 4.0-mm shaver (Torpedo; Arthrex), the important anatomic landmarks may be easily identified (Fig 2), including the ankle joint, subtalar joint, and FHL tendon.

The FHL tendon must be debrided and mobilized carefully by releasing any adhesions, as well as its proximal fascia. In case of difficulty in identifying the tendon, passive plantar flexion and dorsiflexion of the hallux may assist in the recognition of the FHL tendon and muscle belly. It is of utmost importance to avoid working on the medial aspect of the tendon because of the proximity to the medial-lying neurovascular structures (tibial nerve and posterior tibial artery).^1^^,^^7^

**Fig 3:**
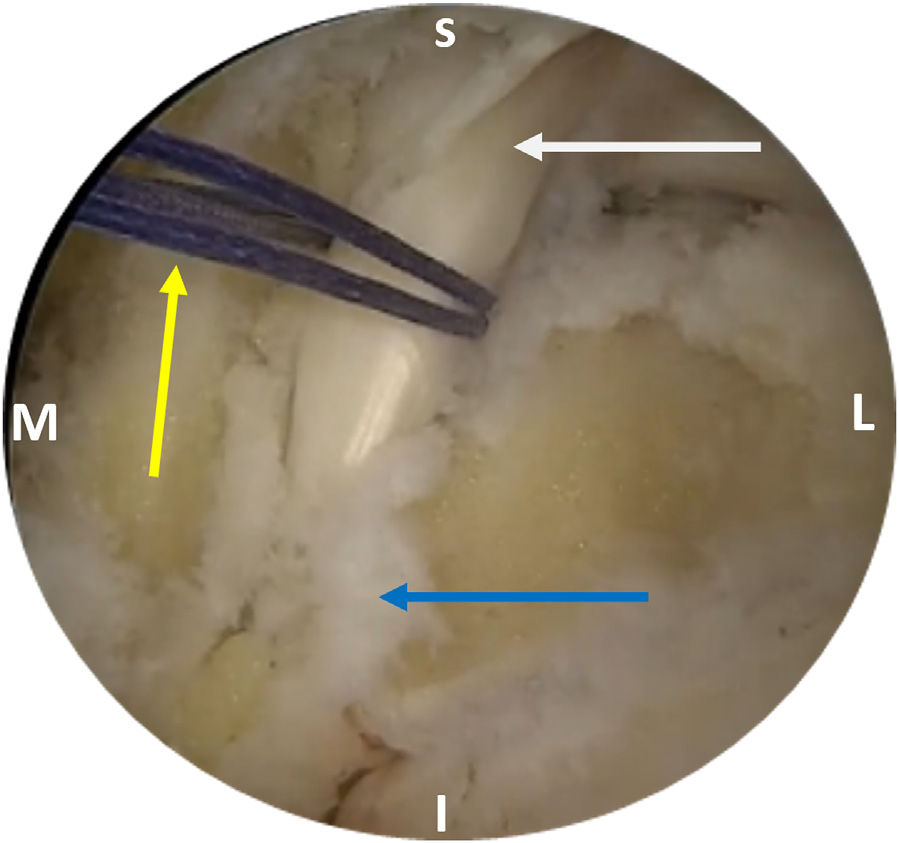
A No. 2-0 Vicryl suture (yellow arrow) is passed around the tendon and is locked in a loop fashion to apply traction and ensure a greater length of the harvested flexor hallucis longus (FHL) tendon. The suture is inserted through the posteromedial (working) portal, and the arthroscope is inserted through the posterolateral (viewing) portal. The white arrow indicates the FHL tendon, and the blue arrow indicates the entrance of the FHL tendon into its fibro-osseous sheath. (I, inferior; L, lateral; M, medial; S, superior.)

**Fig 4:**
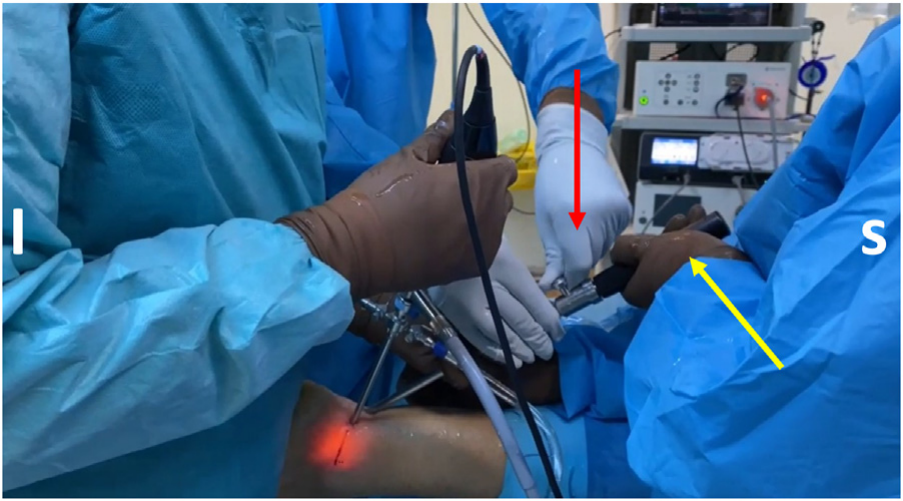
Flexor hallucis longus (FHL) tendon harvesting. A common hamstring tendon stripper is inserted through the posteromedial portal and, after grasping the tendon, is guided into the fibro-osseous canal. The assistant on the left cuts the FHL with the integrated cutting mechanism of the tendon stripper (red arrow), while a second assistant, on the right, holds the stripper steady (yellow arrow) inside the fibro-osseous sheath. (I, inferior; S, superior.)

**Fig 5:**
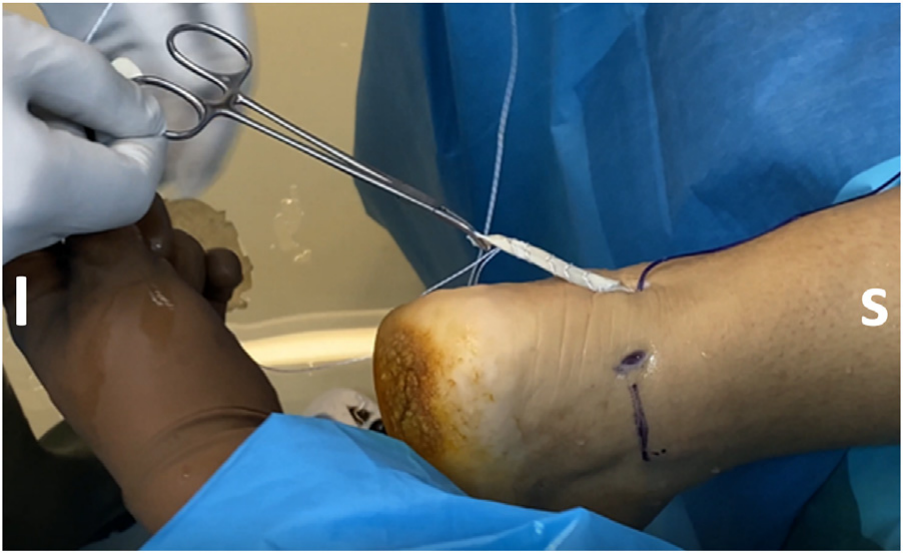
After flexor hallucis longus (FHL) tendon harvesting, a stay suture is applied on the FHL tendon stump in a Krackow fashion. The suture should be strong enough to withstand the tension that will be applied during tendon fixation. (I, inferior; S, superior.)

**Fig 6:**
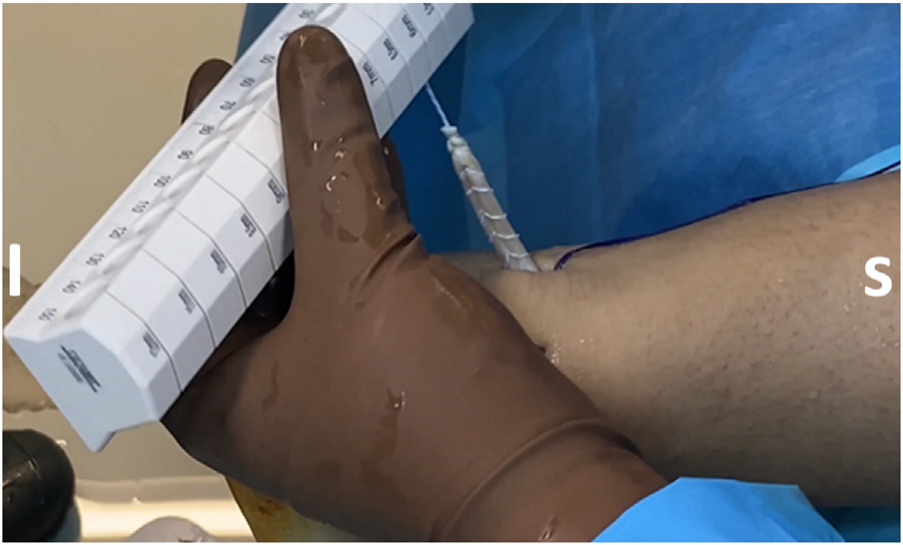
The flexor hallucis longus (FHL) tendon width is measured after harvesting. In most of our patients, the FHL tendon width is approximately 7 mm. (I, inferior; S, superior.)

After adequate mobilization, the next step is FHL tendon harvesting. It is important to preserve the interconnection of the FHL to the flexor digitorum longus at the master knot of Henry (MKH) to maintain the residual hallux flexion power.^8^ Thus, the tendon should be preferably cut in zone 1 (posterior to the ankle) or zone 2 (from the FHL entrance into its fibro-osseous sheath, under the sustentaculum tali, up to the MKH).^9^ In most cases, a minimum tendon length of 15 to 20 mm is required.^1^^,^^7^ A towing suture is first loaded onto a mosquito clamp; it is then inserted through the posteromedial portal and applied in a loop fashion around the tendon to perform traction (Fig 3). A common hamstring tendon stripper is inserted through the same portal and, after grasping the tendon, is guided into the fibro-osseous canal. Forceful stripper thrusting into the canal increases the risk of compressing the tibial nerve, especially if the ankle is positioned in dorsiflexion.^9^ Additionally, the MKH and the medial plantar nerve are jeopardized if the stripper is pushed to maximum length into the canal.^9^ After the stripper is inserted into the canal, the ankle is plantar flexed and the first metatarsophalangeal and interphalangeal joints are also flexed if increased length of the tendon graft is required. At this point, the integrated cutting mechanism of the stripper is used for FHL retrieval (Fig 4). The FHL tendon is withdrawn from the posteromedial portal, and a stay suture (Fiberloop; Arthrex) is applied in Krackow fashion (Fig 5). Then, the diameter of the FHL tendon is measured (Fig 6).

**Fig 7:**
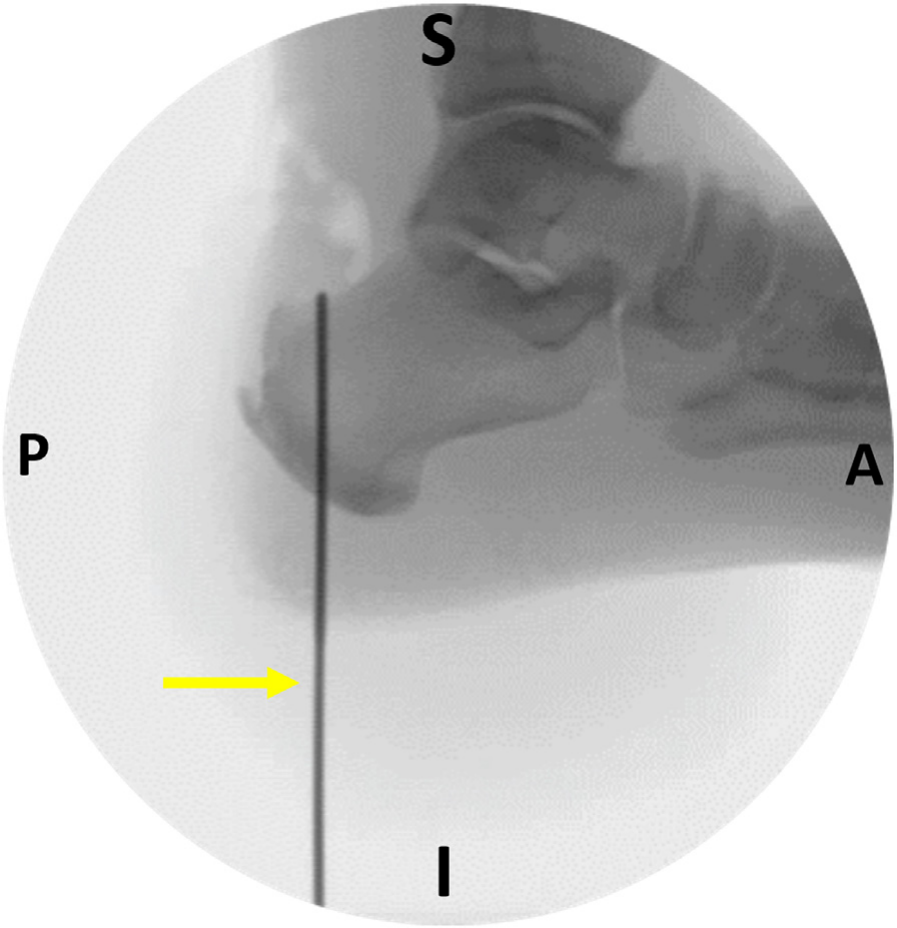
Image intensifier screenshot. Under fluoroscopic guidance, a guidewire (yellow arrow) is placed as posterior and medial as possible into the calcaneus in a retrospective fashion. (A, anterior; I, inferior; P, posterior; S, superior.)

**Fig 8:**
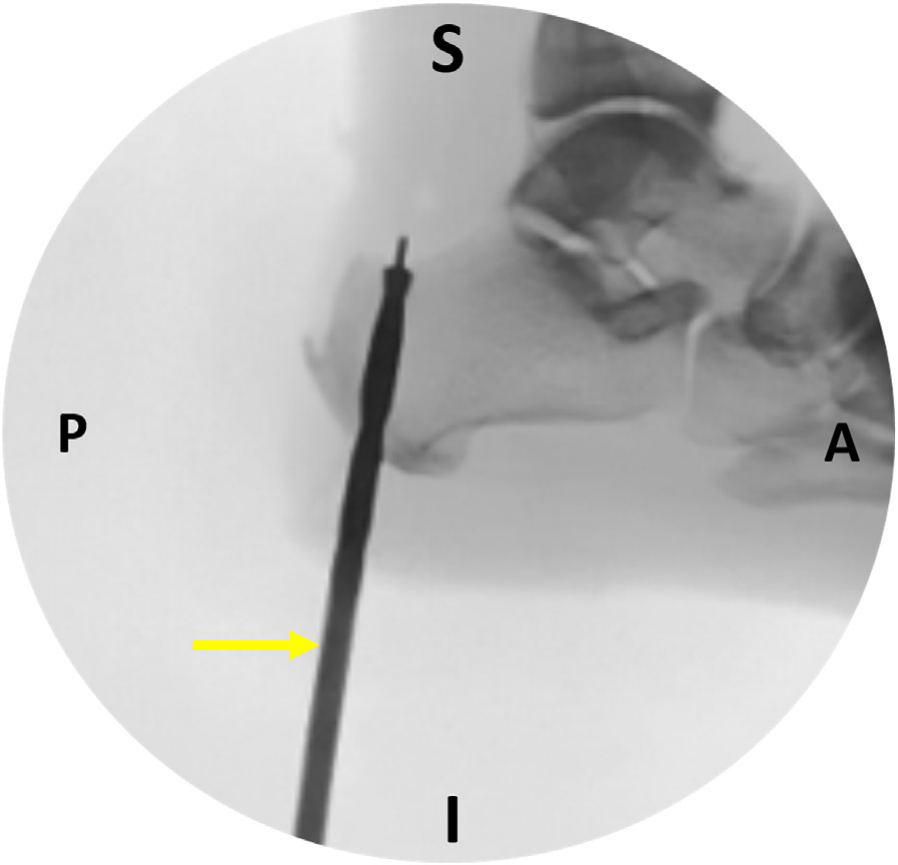
Image intensifier screenshot. Under fluoroscopic guidance, the calcaneal tunnel is drilled. The yellow arrow indicates the drill loaded onto the guidewire. (A, anterior; I, inferior; P, posterior; S, superior.)

**Fig 9:**
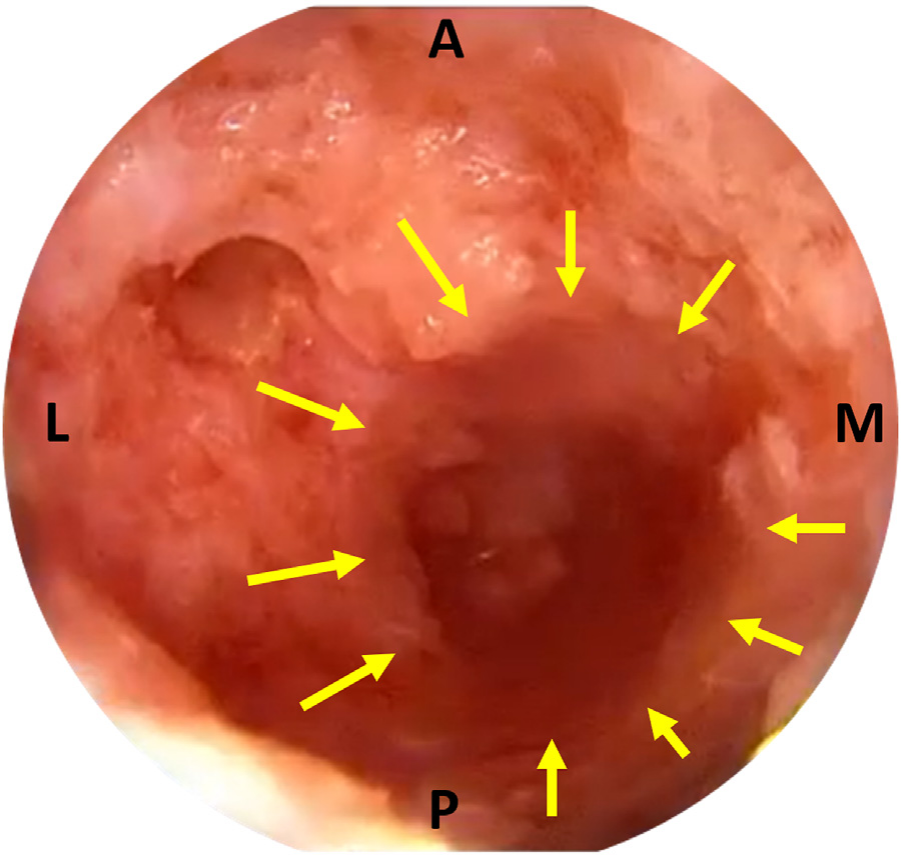
The arthroscope is placed inside the distal aspect of the calcaneal tunnel, through the plantar incision. Direct scoping of the calcaneal tunnel is performed (yellow arrows), and its integrity is ensured. (A, anterior; L, lateral; M, medial; P, posterior.)

The next step is to establish the calcaneal tunnel. The diameter of the tunnel is usually 1 mm greater than the diameter of the harvested FHL tendon. Under fluoroscopic guidance, a guidewire is placed in the calcaneus in retrospective fashion (Fig 7). An anterior cruciate ligament guide may be used to facilitate the correct guidewire placement. The optimal position of the guidewire is as medial and posterior as possible, achieving the maximum biomechanical advantage.^1^^,^^7^^,^^10^ A cannulated drill is placed over the guidewire, and a plantar stab skin incision facilitates drill passage. Calcaneus drilling is checked under fluoroscopy (Fig 8). Direct scoping through the plantar incision may also be performed to ensure the integrity of the tunnel walls (Fig 9). Far posterior tunnel positioning increases the risk of blowout.

**Fig 10:**
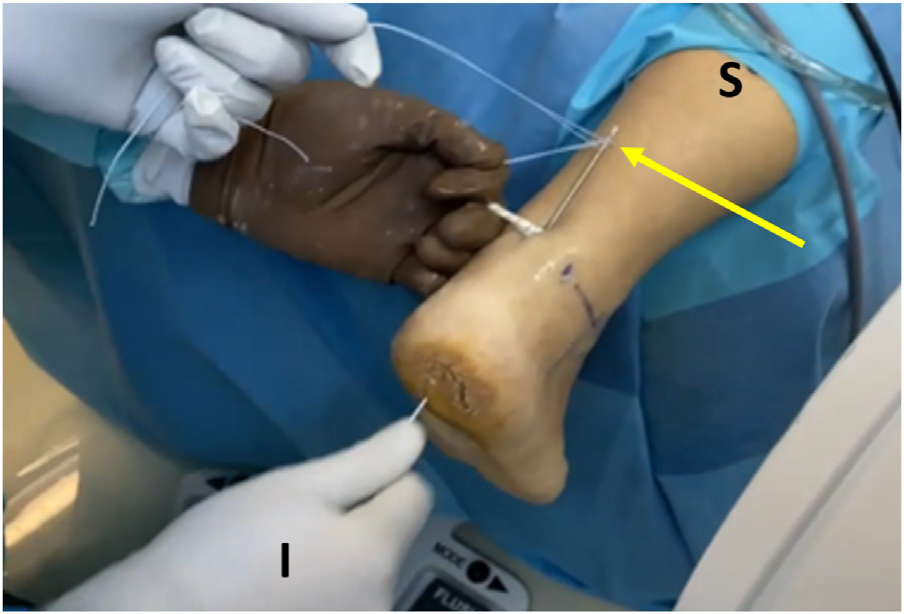
The stay suture of the flexor hallucis longus tendon stump is passed through the tunnel, from superior to inferior, with the assistance of a guidewire with an eyelet (yellow arrow). (I, inferior; S, superior.)

**Fig 11:**
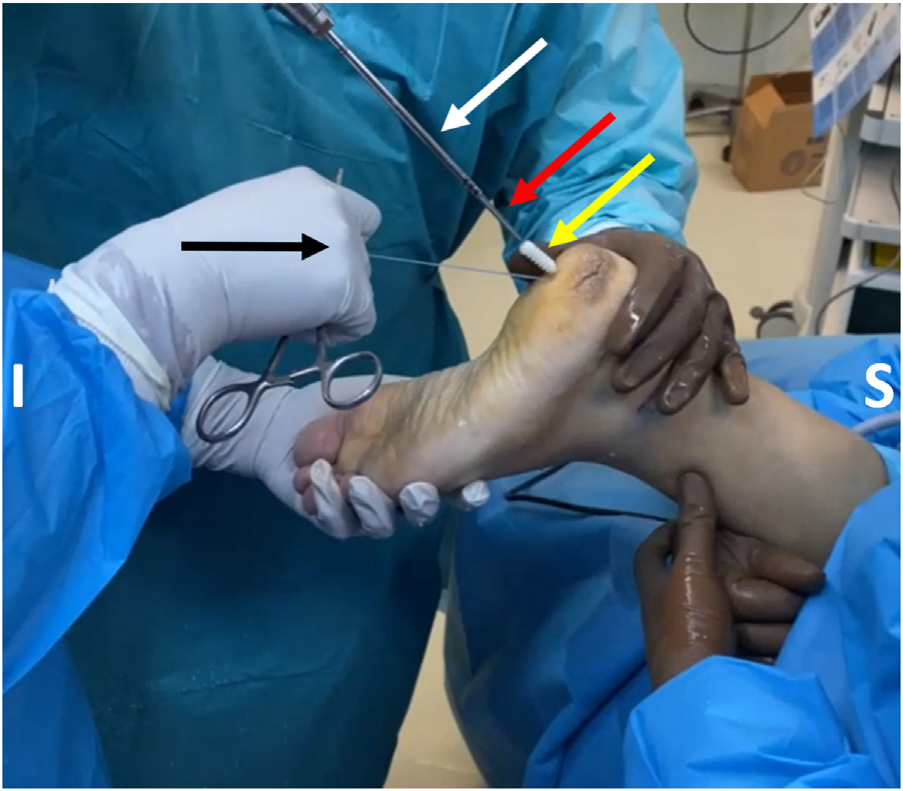
An interference screw (yellow arrow) is loaded onto a screwdriver (white arrow) and is guided into the tunnel with the assistance of a guidewire (red arrow). At this point, tension must be applied on the stay sutures (black arrow) and the patient’s ankle should be passively plantar flexed. (I, inferior; S, superior.)

**Fig 12:**
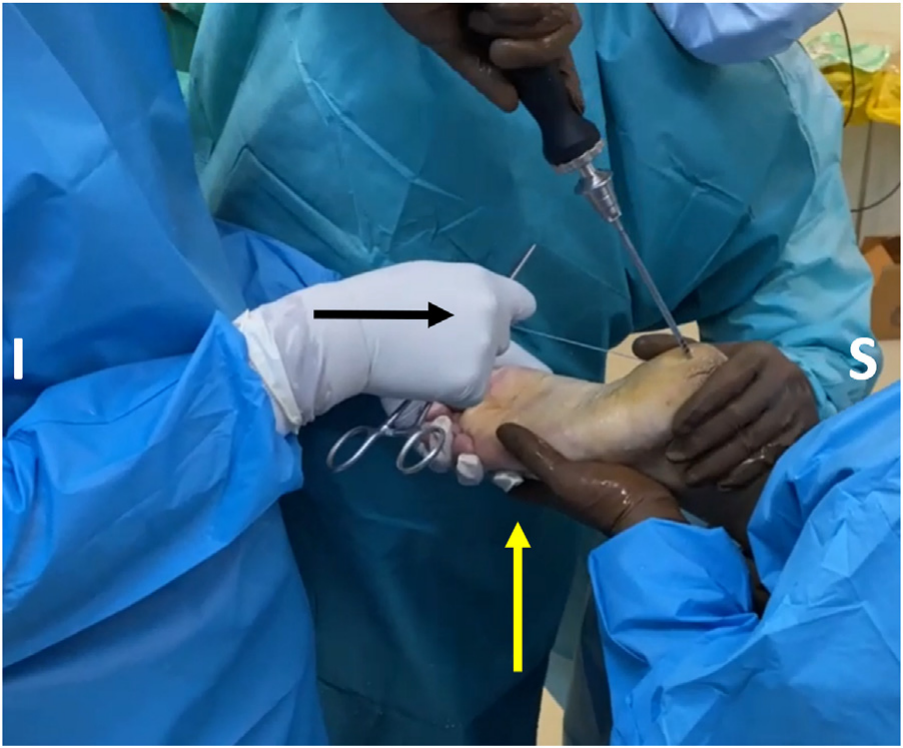
The assistant on the left pulls the stay suture of the flexor hallucis longus stump (black arrow) to ensure fixation under tension. The assistant on the right holds the ankle steady in plantar flexion (yellow arrow). The advancement of the screw is considered adequate if turning of the screwdriver becomes progressively harder combined with the characteristic creaking sound. In addition, to avoid irritation during ambulation, the screw should not be proud distally. (I, inferior; S, superior.)

After tunnel drilling, the FHL tendon must be fixated. The tendon is placed into the tunnel using a 2.4-mm guidewire with an eyelet, which is inserted into the plantar aspect of the tunnel and exits through the posteromedial portal (Fig 10). The stay sutures at the free end of the tendon are passed through the eyelet, and the guidewire is pulled out of the plantar aspect of the tunnel. The sutures' pulling guides the tendon into the tunnel. The passage of the tendon and its presence in the tunnel may also be checked through direct scoping. The ankle is placed in plantar flexion, and a screw guidewire is placed into the plantar aspect of the calcaneal tunnel, in front of the FHL tendon (Fig 11). A bioabsorbable interference screw (BioComposite; Arthrex) or metal cannulated interference screw is placed into the tunnel, guided by the screw guidewire, with the FHL under tension (Fig 12).

**Fig 13:**
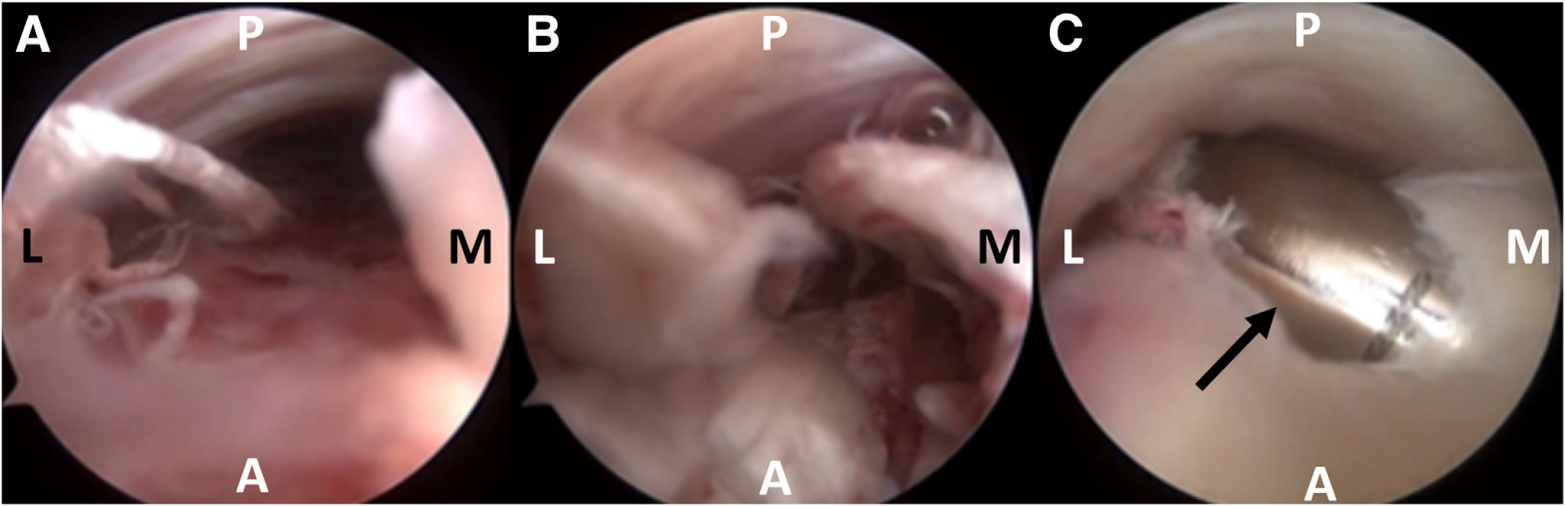
Confirmation of the rupture may be performed with direct Achilles tendon endoscopy. First, the trocar is used to create a working space between the paratenon and the subcutaneous tissue. Meticulous manipulations are required to avoid skin perforation. The arthroscope is introduced through the posterolateral portal and is guided proximally to visualize the proximal stump. (A) The arthroscope is slowly withdrawn distally until some degenerated fibers are seen, indicating proximity to the rupture site. (B) The rupture site is observed as a jumble of degenerated fibers. (C) A shaver (black arrow) may be introduced through the posteromedial portal and used to debride the degenerated fibers to increase bleeding and, consequently, the healing potential of the ruptured stumps. Caution is advised to debride only the rupture site and avoid damage to the subcutaneous soft tissue or the skin. Performing this step is optional and is left to the surgeon’s preference. (A, anterior; L, lateral; M, medial; P, posterior.)

Finally, the AATR may be confirmed endoscopically. A trocar may be used to create the necessary space between the paratenon and the subcutaneous tissue, and the arthroscope is then introduced into the rupture. At this point, the torn AT’s stumps may be debrided, increasing local bleeding and facilitating healing (Fig 13). After the completion of debridement, the portals and the plantar incision are closed with nonabsorbable sutures, and a sterile dressing and below-knee cast in the equinus position are applied for 2 weeks. After cast removal, a walking boot is placed in the equinus position and is gradually moved into neutral position during a 4-week period.

## Discussion

Endoscopic FHL tendon transfer is a promising arthroscopic technique for AATR management. It combines the advantages of biological and biomechanical AT enhancement, while it avoids major surgical trauma. The FHL tendon represents an ideal candidate for tendon transfer in AT ruptures. At first, the FHL fires “in phase” with the triceps surae, while its vector of force is parallel to the vector of force transmitted to the calcaneus through the intact AT. Furthermore, the more posterior positioning of the FHL tendon at the calcaneus increases the lever arm and generates greater ankle flexion torque. The transferred FHL tendon also acts as an internal brace, ensuring the AT stumps’ approximation.^11^ The main tips and pitfalls of our technique are highlighted in Table 1.

**Table 1:** Tips and Pitfalls of Endoscopic FHL Transfer

Tips	Pitfalls
Posterior translation of the FHL muscle belly is facilitated by adequate release of the proximal FHL fascia.	Cautery should be used only when necessary; a rising saline solution temperature may affect nearby nerve function.
FHL muscle-tendon unit bending is avoided by adequate release of the proximal FHL fascia.	The surgeon should always work on the lateral side of the FHL to avoid injury to the neurovascular bundle.
Tendon harvesting may be performed with a percutaneous blade or arthroscopic scissors.	The surgeon should avoid forceful stripper thrusting distally during tendon harvesting.
The tunnel position should be as medial and posterior as possible.	The surgeon should be sure to obtain an FHL tendon length of at least 15-20 mm and consider ankle and hallux passive flexion during FHL tendon harvesting.
Either a BioComposite screw or metal screw may be used.	To avoid tunnel blowout, drilling must not be performed too posteriorly or medially.
If the calcaneal bone is soft (e.g., owing to osteoporosis), the use of a wider screw should be considered.	A proud plantar-lying interference screw may irritate the plantar fat pad and the postoperative scar during ambulation.

FHL, flexor hallucis longus.

The described operation not only produces great biomechanical impact on the hindfoot but also has an important effect on the AT’s healing potential. The posterior translation of the FHL results in closer proximity of the AT to the FHL muscle belly, which is rich in blood vessels. The FHL muscle-tendon junction lies distal enough to ensure the AT-FHL muscle belly proximity in non-insertional AT ruptures. This proximity may be facilitated by the FHL proximal fascia release. Another biological advantage is the minimally invasive approach, which preserves the paratenon and does not require long skin incisions, reducing the infection risk and wound healing complications.^8^

In the described technique, it is of note that 2 steps have not been described so far. It is our preference to drill the calcaneal tunnel retrospectively rather than through the posteromedial portal, which is mainly reported. Furthermore, the interference screw is placed in the same fashion. We believe it is easier for the surgeon to perform these steps in this way, while attention should be paid to plantar screw prominence, which could lead to fat pad irritation. Additionally, we prefer to debride the torn AT stumps through direct scoping. We advocate that degenerative tissue debridement and local bleeding may improve the AT’s healing potential. The anticipated donor-site morbidity would be the reduction of hallux flexion power, resulting in push-off weakness and impaired ambulation. However, the loss of hallux flexion power seems to be minimal if the FHL tendon is harvested proximal to the MKH.^1^^,^^4^^,^^7^^,^^8^^,^^11^

The main advantages and disadvantages of the described procedure are summarized in Table 2. The obvious advantage of endoscopic FHL tendon transfer, compared with the open technique, is that it is minimally invasive. Hence, it is safe and reliable even in high-risk patients.^4^ Nevertheless, endoscopic FHL tendon transfer is technically demanding and should be performed by experienced surgeons who are familiar with the hindfoot anatomy. Adequate prior cadaveric practice is also suggested to avoid technical errors.^4^^,^^6^

**Table 2:** Advantages and Disadvantages of Endoscopic FHL Transfer

Advantages
Endoscopic FHL transfer is a safe and reliable treatment option.
The FHL muscle belly, rich in vessels, is placed in proximity to the anterior surface of the ruptured Achilles tendon, enhancing healing potential.
The transferred tendon acts as an internal brace, decreasing Achilles tendon stump withdrawal.
Calcaneoplasty is not a necessity if drilling is performed in a retrograde manner.
The paratenon remains intact, preserving vascularity.
With small incisions and minimal trauma, the technique is a viable option for high-risk patients.
Minimal donor-side morbidity is reported; the decrease in hallux plantar flexion power is not significant.
Disadvantages
The operation is technically demanding.
The technique requires adequate surgical experience and familiarity with hindfoot endoscopy; some authors suggest prior cadaveric practice.^4^
The technique requires more special equipment (image intensifier and arthroscopy-related equipment) than minimally invasive Achilles tendon repair and open repair techniques.
The operative time is increased compared with minimally invasive Achilles tendon repair and open repair.

FHL, flexor hallucis longus.

## Supplementary Data

ICMJE author disclosure forms

Video 1 Endoscopic flexor hallucis longus (FHL) transfer. The patient is placed in the prone position. A posteromedial portal and a posterolateral portal are established. After careful soft-tissue debridement, the FHL muscle-tendon unit is identified, and its proximal fascia is released. A towing suture is applied in a loop fashion around the FHL tendon to perform traction. FHL tendon harvesting is performed with a hamstring tendon stripper, and the free end of the tendon is withdrawn from the posteromedial portal. A stay suture is applied, and its diameter is measured. Under fluoroscopy, a guidewire is placed retrospectively through the calcaneus as posterior and medial as possible. A cannulated drill is used for calcaneus tunnel establishment. The stay suture is passed through the tunnel with an eyelet guidewire. The FHL tendon is introduced into the tunnel by pulling the suture. The FHL is fixated with a bioabsorbable interference screw, which is introduced in retrospective fashion. The ankle should be plantar flexed during FHL fixation. Achilles tendon rupture is confirmed with direct scoping, and the tendon stumps are debrided. The last video slide presents the postoperative rehabilitation protocol.
